# CT-based deep learning signatures associated with transcriptomic heterogeneity and combined with nutritional biomarkers improve prediction of 3-year overall survival in esophageal squamous cell carcinoma

**DOI:** 10.1186/s13244-025-02189-x

**Published:** 2026-01-26

**Authors:** Jianye Jia, Yahui Cheng, Jiahao Wang, Genji Bai, Lei Han, Lixue Xu, Yantao Niu

**Affiliations:** 1 Department of Radiology, Beijing Friendship Hospital, Capital Medical University, Beijing, China; 2 Department of Radiology, Nanjing Pukou People’s Hospital, Liangjiang Hospital, Southeast University, Nanjing, PR China; 3 Department of Medical Imaging Center, The Affiliated Huaian NO.1 People’s Hospital of Nanjing Medical University, Huaian, PR China; 4 Department of Medical Imaging, Huaian Hospital Affiliated to Xuzhou Medical University, Huaian, China

**Keywords:** Enhanced-CT, Deep learning, Esophageal squamous cell carcinoma, Overall survival, Tumor microenvironment

## Abstract

**Objective:**

Deep learning signatures (DLS) extracted from CT images can noninvasively reflect tumor heterogeneity and have shown promise in prognostic modeling for esophageal squamous cell carcinoma (ESCC). To develop and validate a CT-based DL model combined with nutritional biomarkers to predict 3-year overall survival (OS) in ESCC, and to investigate transcriptomic differences between DLS-based risk groups.

**Materials and methods:**

This retrospective multicenter study included 662 postoperative ESCC patients from three hospitals and 16 additional patients from The Cancer Genome Atlas (TCGA). DL features extraction from CT images based on the Crossformer architecture. Skeletal muscle index was measured at the L3 vertebra to assess low skeletal muscle mass (LSMM). Cox regression was used to build clinical, DL, and combined models. Model performance was evaluated using the concordance index (*C*-index). Transcriptomic analysis of the TCGA cohort was performed to identify metabolic pathway differences between DLS-based risk groups.

**Results:**

The DL model achieved a *C*-index of 0.743 (95% CI: 0.683–0.803) in the internal validation cohort and 0.692 (95% CI: 0.576–0.809) in the external cohort. Pathological T and N stages, Neuroaggression, Vascular invasion, and LSMM were identified as independent clinical predictors. The combined model achieved a *C*-index of 0.753 (95% CI: 0.697–0.808) internally and 0.725 (95% CI: 0.613–0.838) externally. DLS–based risk stratification revealed significant differences in metabolic activity between groups, supporting its biological relevance.

**Conclusion:**

The combined model enables preoperative OS prediction in ESCC. DLS–based stratification reflects transcriptomic metabolic heterogeneity and enhances the biological interpretability of imaging features.

**Critical relevance statement:**

This study developed a CT-based DLS and combined it with nutritional markers for prognostic modeling in ESCC. Transcriptomic analysis of DLS-based groups revealed metabolic heterogeneity, enhancing the biological interpretability of the DL model.

**Key Points:**

- A combined DLS and nutritional model enables individualized preoperative survival prediction in ESCC.
- DLS-based risk groups defined by the DLS exhibited transcriptomic differences in key metabolic pathways, revealing biological underpinnings of imaging-based phenotypes.
- Attention map visualization revealed consistent spatial focus on morphologically distinct tumor regions, enhancing the interpretability of deep learning predictions.

**Graphical Abstract:**

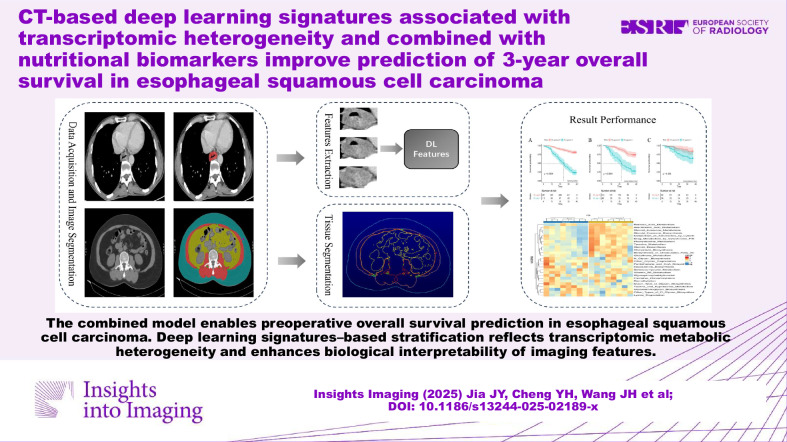

## Introduction

Esophageal cancer is the sixth leading cause of cancer-related death worldwide, with esophageal squamous cell carcinoma (ESCC) being the most common histological subtype and associated with poor prognosis [1]. Despite surgical and perioperative improvements, long-term survival remains suboptimal, with 5-year overall survival (OS) rates under 20% and 3-year OS below 50% in many cases [2]. Current prognostic assessments rely primarily on postoperative pathological parameters such as TNM stage, differentiation, and vascular invasion, which fail to capture inter-patient biological heterogeneity [3, 4]. Significant survival variation among patients with identical pathological stages underscores the need for preoperative tools that support individualized risk stratification and treatment planning [5].

Recent studies have recognized malnutrition and low skeletal muscle mass (LSMM)—characterized by progressive skeletal muscle loss—as key predictors of poor outcomes in ESCC [6, 7]. Early assessment of nutritional status may guide prehabilitation and reduce perioperative complications. LSMM has been validated as an independent prognostic factor across multiple solid malignancies, including ESCC [8]. Yet, many DL-based survival models overlook this dimension. DL enables the extraction of high-dimensional features from routine CT images, offering a noninvasive approach to assess tumor morphology and heterogeneity [9–11]. In ESCC, DL models have shown potential for outcome prediction, but most existing models lack integration of key preoperative factors such as nutritional risk and LSMM, thereby limiting their applicability in real-world clinical settings [12, 13]. To address these gaps, we propose a combined model that integrates CT-based DL features with nutritional indicators, including LSMM, for preoperative prediction of 3-year OS in ESCC. This approach aims to bridge imaging phenotypes with patient-level clinical risk, enhancing the robustness and clinical utility of predictive tools, particularly when incorporating validated clinical markers such as LSMM.

Furthermore, tumor metabolic reprogramming plays a crucial role in cancer progression and treatment resistance [14, 15]. Metabolic heterogeneity influences the tumor microenvironment and is closely associated with survival, immune infiltration, and therapy response [16, 17]. Exploring transcriptomic differences between DLS-based risk groups may reveal the biological foundations of phenotypes. This study not only develops a prognostic model but also elucidates the metabolic basis of its DLS through transcriptomic analysis, supporting both interpretability and clinical translation.

## Materials and methods

### Patient population

**Fig. 1:**
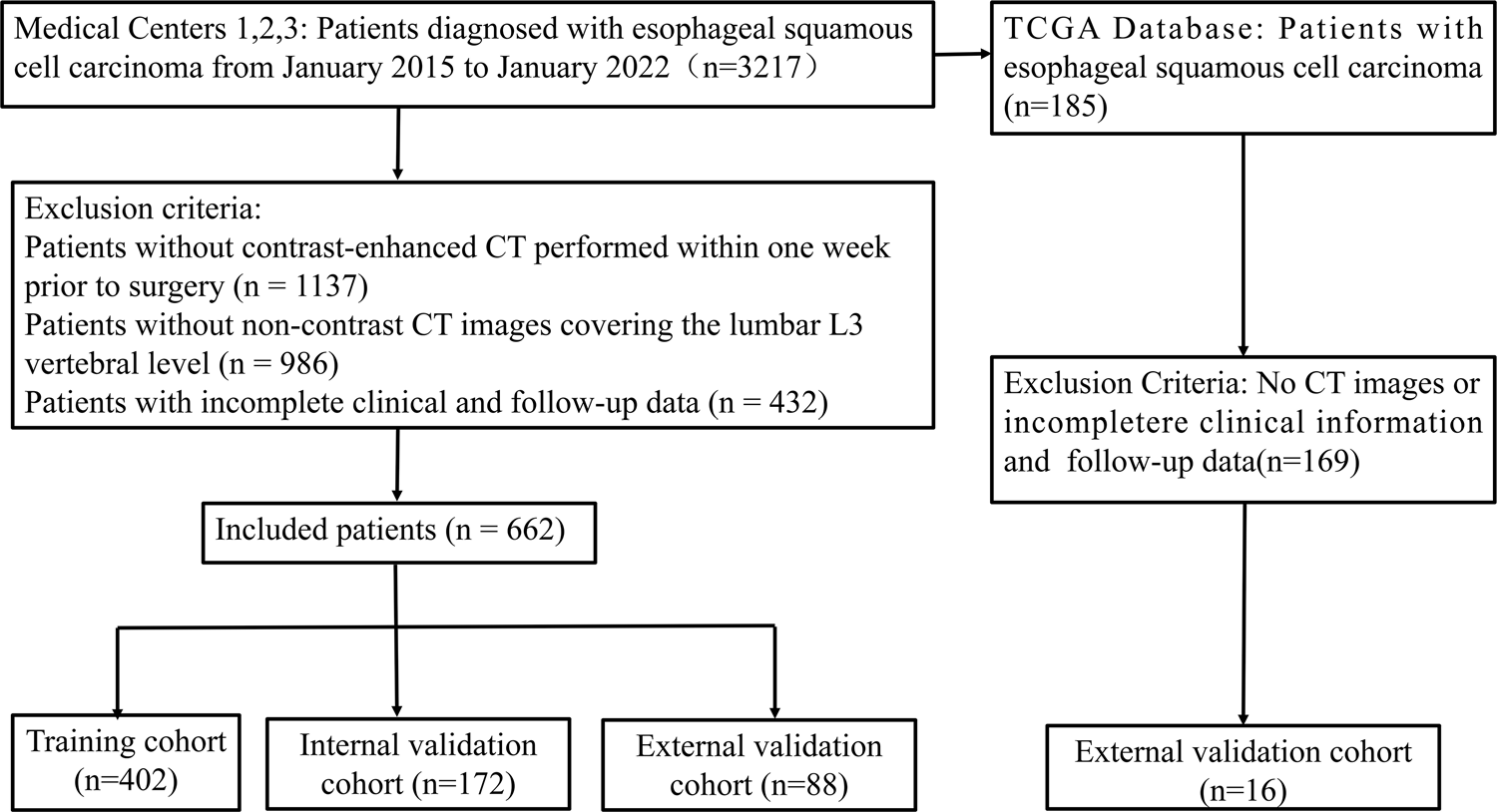
Flow chart of the patients’ recruitment pathway

This was a multicenter retrospective study that included a total of 678 patients with ESCC from three medical centers and the Cancer Genome Atlas (TCGA) database between January 2015 and January 2022. Among them, 574 patients from Center 1 and Center 2 were randomly divided into a training cohort (*n* = 402) and an internal validation cohort (*n* = 172) at a ratio of 7:3. Eighty-eight patients from Center 3 were assigned to an external validation cohort. In addition, 16 ESCC patients from the TCGA database were included for the analysis of the biological mechanisms underlying the DL model (patient selection flowchart shown in Fig. 1). Clinical information and transcriptomic data of TCGA patients were obtained from the GDC Data Portal (https://portal.gdc.cancer.gov/), and CT images were retrieved from The Cancer Imaging Archive (TCIA; https://www.cancerimagingarchive.net/). The inclusion criteria were as follows: (1) postoperative pathological confirmation of ESCC; (2) completion of contrast-enhanced CT within one week before surgery, with acceptable image quality; (3) availability of non-contrast CT images within one week before surgery, including the L3 vertebral level; and (4) complete clinical and follow-up information. Exclusion criteria were as follows: (1) receipt of any form of antitumor therapy before surgery; (2) presence of other malignancies or distant metastases; and (3) poor-quality CT images or incomplete clinical/follow-up data. The study received approval from the Ethics Committee of the Affiliated Huai’an First Hospital of Nanjing Medical University, and informed consent was waived. Ethical approval number: KY-2022-045-01.

### Clinical and follow-up data of patients

Baseline clinical characteristics, postoperative pathology, and survival data were collected for all patients. Preoperative nutritional status was evaluated by assessing skeletal muscle index (SMI) at the L3 vertebral level using CT images. LSMM was defined using sex-specific SMI thresholds, and its association with prognosis was analyzed [18, 19] (detailed definitions in Supplementary Material 1). The primary endpoint was 3-year OS, defined as the interval from the date of surgical resection to death from any cause. Patients were followed for up to 3 years postoperatively or until December 2024, whichever came first. For patients without events, survival data were censored at the last confirmed follow-up within this period. Follow-up data were obtained via electronic medical records and telephone interviews.

### CT image acquisition and DL features extraction

All CT scans were acquired using Siemens SOMATOM Force and SOMATOM Definition scanners (detailed parameters in Supplementary Materia 2). Venous-phase images were preprocessed via voxel resampling, gray-level discretization, and normalization. Two experienced thoracic radiologists manually segmented 3D regions of interest (ROIs) on axial CT images using ITK-SNAP (v3.8.0, http://www.itksnap.org), with adjustments in coronal and sagittal views. Crossformer extracted multiscale features via convolution and attention. PCA reduced them to 256-dimensional representations for modeling.

### Differential gene expression and pathway functional analysis

**Fig. 2:**
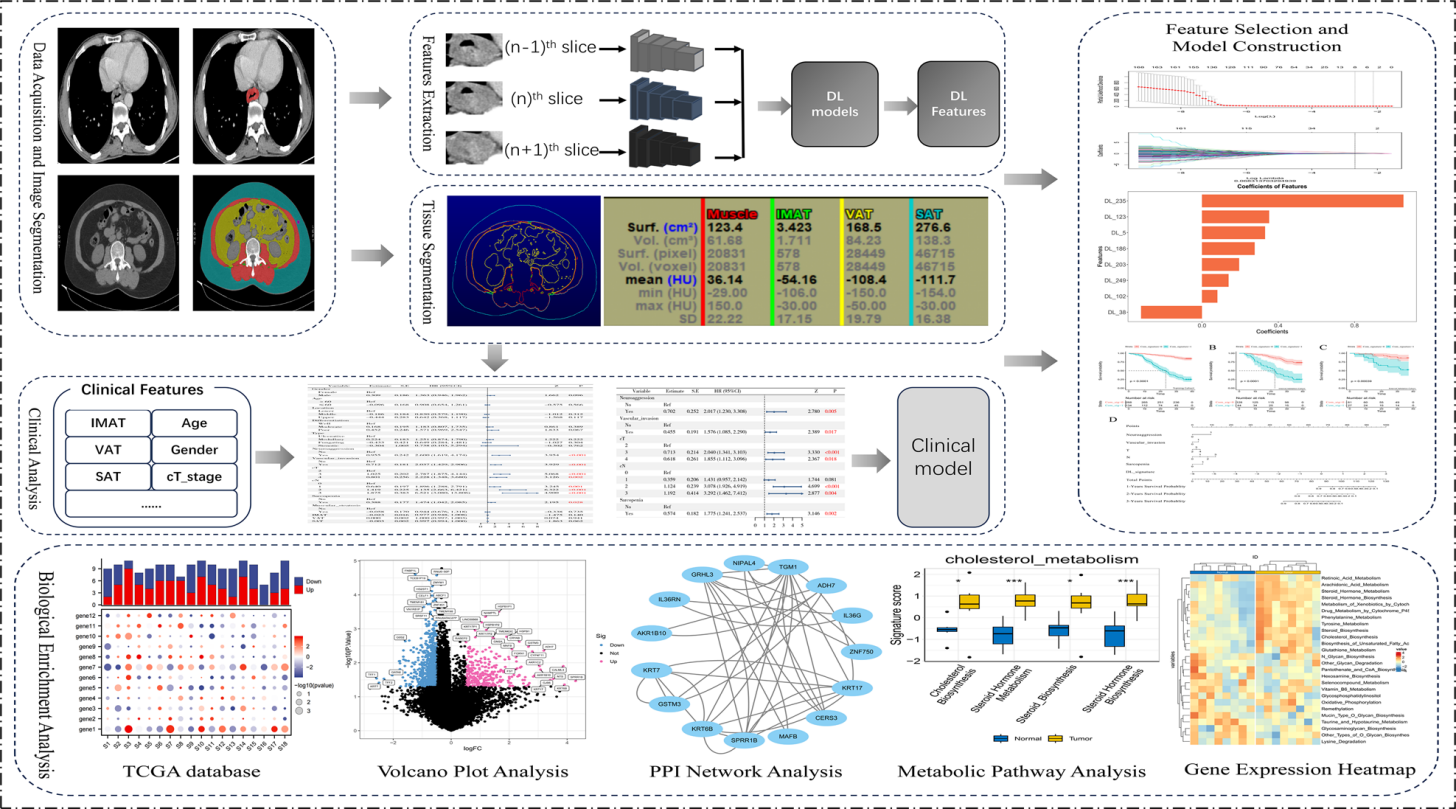
Overview of study workflow

Differentially expressed genes (DEGs) between high- and low-risk groups were identified using the “limma” package and visualized via volcano plots and clustering heatmaps. A protein–protein interaction (PPI) network was constructed to identify key functional modules. To explore metabolic heterogeneity, single-sample gene set enrichment analysis (ssGSEA) was performed on curated metabolism-related gene sets, including pathways like cholesterol biosynthesis, steroid metabolism, glutathione metabolism, and oxidative phosphorylation. Enrichment score distributions were compared using Wilcoxon tests, and Spearman correlation was applied to assess associations between the DLS and pathway activity, revealing how imaging features may reflect underlying metabolic states (Fig. 2).

### Statistical analysis

Statistical analyses were performed using R (v4.1.3; www.R-project.org) and SPSS (v25.0; IBM). Continuous variables were compared using independent *t*-tests or Mann–Whitney *U*-tests, as appropriate. Univariate and multivariate Cox regression analyses identified independent prognostic factors. Least absolute shrinkage and selection operator (LASSO)-Cox regression (“glmnet”), nomogram construction and calibration (“rms”), and Kaplan–Meier (KM) survival analysis were conducted in R. Risk stratification was based on DL, clinical, and combined signatures derived from the respective Cox models, with optimal cutoffs determined in the training set using the survminer R package and applied to the validation and external cohorts. Model performance was evaluated using the concordance index (*C*-index), calibration curves, and decision curve analysis (DCA) in both training and validation cohorts.

## Results

### Patient clinical characteristics

A total of 662 ESCC patients from three medical centers were included in this study. Among them, 574 patients were from Center 1 and Center 2, with 212 deaths recorded (36.90%). Center 3 included 88 patients, of whom 20 (22.70%) died during follow-up. Additionally, 16 patients from the TCGA database were included, with 9 (56.30%) deaths observed during the follow-up period. Baseline characteristics of patients from the three medical centers are summarized in Table 1. The median follow-up time was 50.1 months (interquartile range: 43.3–62.3).

**Table 1:** Characteristics of ESCC patients in training, internal validation, and external validation cohorts

Variable	Training cohort (*n* = 402)	Internal validation cohort (*n* = 172)	External validation cohort (*n* = 88)	*p*-value
Gender, *n* (%)				0.532
Male	131 (32.59)	52 (30.23)	19 (21.59)	
Female	271 (67.41)	120 (69.77)	69 (78.41)	
Age, *n* (%)				0.214
≤ 65	228 (56.72)	86 (50.00)	39 (44.32)	
> 65	174 (43.28)	86 (50.00)	49 (55.68)	
Location, *n* (%)				0.481
Upper	60 (14.93)	22 (12.79)	8 (9.09)	
Middle	225 (55.97)	94 (54.65)	46 (52.27)	
Lower	117 (29.10)	56 (32.56)	34 (38.64)	
Differentiation, *n* (%)				0.301
Well	126 (31.34)	57 (33.14)	13 (14.77)	
Moderate	214 (53.23)	91 (52.91)	44 (50.00)	
Poor	62 (15.42)	24 (13.95)	31 (35.23)	
Type, *n* (%)				0.627
Ulcerative	268 (66.67)	108 (62.79)	57 (64.77)	
Medullary	105 (26.12)	52 (30.23)	6 (6.82)	
Fungating	25 (6.22)	7 (4.07)	21 (23.86)	
Stenotic	4 (1.00)	5 (2.91)	4 (4.55)	
Neuroaggression, *n* (%)				0.415
No	369 (94.79)	156 (90.70)	72 (81.82)	
Yes	33 (8.21)	16 (9.30)	16 (18.18)	
Vascular invasion, *n* (%)				0.543
No	320 (79.60)	135 (78.49)	77 (87.50)	
Yes	82 (20.40)	37 (21.51)	11 (12.50)	
pT, *n* (%)				0.334
T2	165 (41.04)	114 (66.28)	29 (32.95)	
T3	170 (42.29)	45 (26.16)	58 (65.91)	
T4	67 (16.67)	13 (7.56)	1 (1.14)	
pN, *n* (%)				0.292
N0	215 (53.48)	91 (52.91)	52 (59.09)	
N1	129 (32.09)	62 (36.05)	29 (32.95)	
N2	48 (11.94)	16 (9.30)	7 (7.95)	
N3	10 (2.49)	3 (1.74)	0 (0.00)	
Therapy				0.501
No	293 (72.89)	140 (81.40)	80 (90.91)	
Yes	109 (27.11)	32 (16.60)	8 (9.09)	
Sarcopenia, *n* (%)				0.478
No	293 (72.89)	123 (71.51)	71 (80.68)	
Yes	109 (27.11)	49 (28.49)	17 (19.32)	
Muscular steatosis, *n* (%)				0.396
No	159 (39.55)	65 (37.79)	41 (46.59)	
Yes	243 (60.45)	107 (62.21)	47 (53.41)	
Height	165.00 (160.00, 170.00)	166.50 (160.00, 170.00)	165.00 (157.75, 170.00)	0.285
Weight	64.00 (57.00, 70.00)	63.00 (57.00, 70.00)	61.00 (55.00, 70.00)	0.311
IMAT, M (Q_1_, Q_3_)	5.92 (3.43, 10.35)	6.47 (3.64, 9.15)	4.51 (3.05, 7.55)	0.352
VAT, M (Q_1_, Q3)	81.53 (47.72, 124.50)	85.03 (50.08, 130.20)	77.60 (46.02, 125.40)	0.489
SAT, M (Q_1_, Q_3_)	84.64 (55.32, 122.55)	111.70 (78.30, 170.07)	76.28 (48.83, 111.65)	0.268

Variables were categorized and are presented as *n* (%)

*M* median, *Q₁* 1st quartile, *Q₃* 3st quartile, *IMAT* intermuscular adipose tissue, *VAT* visceral adipose tissue, *SAT* subcutaneous adipose tissue

### Construction and evaluation of the clinical model

**Fig. 3:**
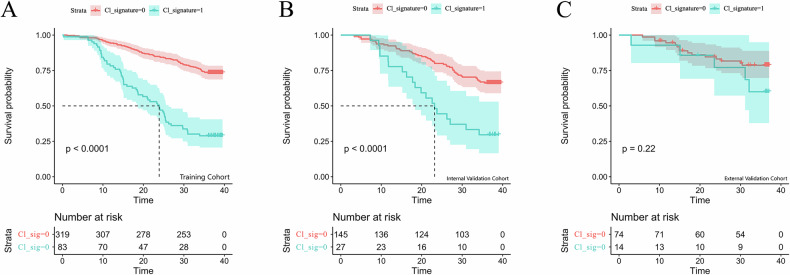
KM survival curves for the clinical model in ESCC patients. **A** Training cohort, (**B**) internal validation cohort, and (**C**) external validation cohort

In the training cohort, clinical and pathological variables were evaluated for their impact on 3-year OS in ESCC patients. Univariate (Supplementary Fig. 1) and multivariate (Table 2) Cox regression analyses identified pathological T stage, pathological N stage, Neuroaggression, vascular invasion, and LSMM as independent prognostic factors. Based on these variables, a clinical prediction model was developed to preoperatively assess the 3-year OS risk in ESCC patients. The model yielded a *C*-index of 0.687 (95% CI: 0.627–0.748) in the internal validation cohort and 0.645 (95% CI: 0.539–0.752) in the external validation cohort. Risk stratification based on the clinical model’s risk scores demonstrated a significant difference in 3-year OS between the high-risk and low-risk groups in both the training and internal validation cohorts, as shown by KM survival curves (*p* < 0.001; Fig. 3).

**Table 2:** Multivariable Cox regression analysis for association with OS

Variable	Estimate	(*S.E*)	HR (95% CI)	*Z*	*p*-value
Neuroaggression					
No	Ref				
Yes	0.702	0.253	2.017 (1.230, 3.309)	2.780	0.005
Vascular invasion					
No	Ref				
Yes	0.455	0.191	1.576 (1.085, 2.290)	2.389	0.017
pT					
2	Ref				
3	0.715	0.228	2.044 (1.306, 3.198)	3.131	0.002
4	0.629	0.497	1.876 (0.709, 4.967)	1.267	0.205
pN					
0	Ref				
1	0.358	0.206	1.431 (0.956, 2.142)	1.741	0.082
2	1.132	0.378	3.102 (1.478, 6.510)	2.993	0.003
3	1.202	0.565	3.326 (1.099, 10.069)	2.126	0.034
Therapy					
Yes	Ref				
No	0.012	0.444	1.012 (0.424, 2.416)	0.027	0.979
LSMM					
No	Ref				
Yes	0.573	0.184	1.774 (1.238, 2.542)	3.122	0.002

### Construction and evaluation of the deep learning model

**Fig. 4:**
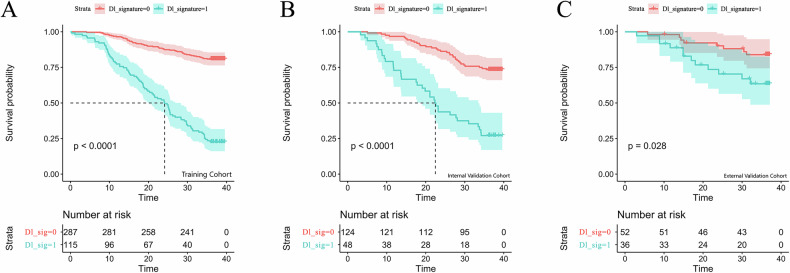
KM survival curves for the Deep learning model in ESCC patients. **A** Training cohort, (**B**) internal validation cohort, and (**C**) external validation cohort

The initial DL features were first reduced using the minimum redundancy maximum relevance algorithm to eliminate redundancy. Subsequently, key features significantly associated with 3-year OS were selected using the LASSO-Cox regression model. At the optimal λ value, eight DL features were identified (Supplementary Fig. 2). These selected features were then weighted by their respective regression coefficients to calculate an individual DLS, which was used to construct the DLS-based prognostic model. This model demonstrated strong predictive performance for 3-year OS in ESCC patients, with a *C*-index of 0.743 (95% CI: 0.683–0.803) in the internal validation cohort and 0.692 (95% CI: 0.576–0.809) in the external validation cohort. Patients were stratified into high-risk and low-risk groups based on the risk score. KM survival analysis revealed a statistically significant difference in 3-year OS between the two groups (*p* < 0.05; Fig. 4).

### Construction and evaluation of the combined model

**Fig. 5:**
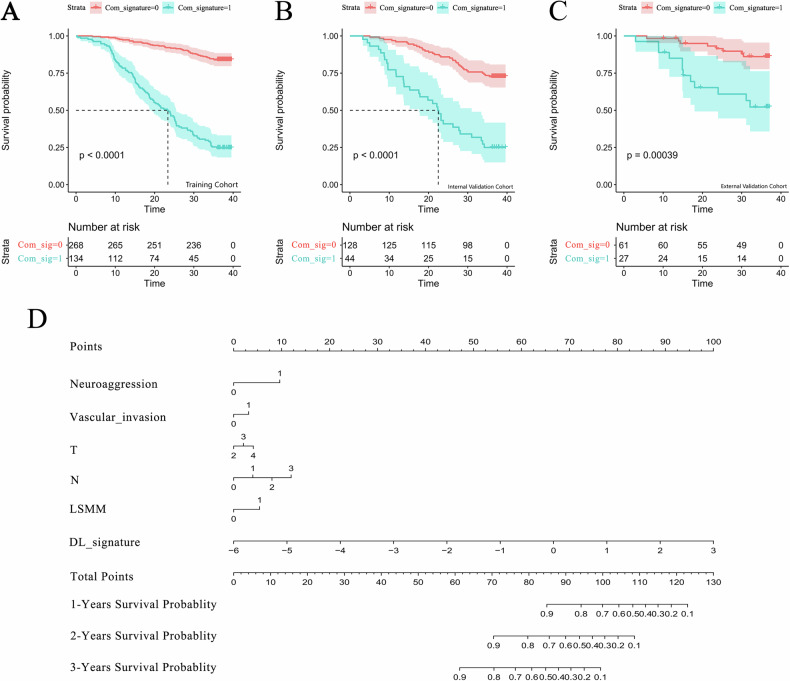
Survival analysis and visualization of the combined model. **A** Training, (**B**) internal validation, and (**C**) external validation cohorts. **D** A nomogram was constructed based on independent clinical factors (T stage, N stage, perineural invasion, vascular invasion, LSMM) and the DLS to predict 1-, 2-, and 3-year OS probabilities

The combined model was implemented as a meta-model: a DL-only Cox model was first trained on DL features selected via LASSO to obtain the DLS (linear predictor). This DLS was then used as a single covariate, together with the significant clinical predictors (T stage, N stage, Neuroaggression, vascular invasion, and LSMM), in a multivariable Cox model to estimate 3-year OS in ESCC. A nomogram was constructed to facilitate individualized risk assessment. The combined model outperformed the clinical and DLS models, achieving *C*-index values of 0.753 (95% CI: 0.697–0.808) and 0.725 (95% CI: 0.613–0.838) in internal and external validation cohorts, respectively (Table 3). KM curves showed significant survival differences between risk groups (*p* < 0.05; Fig. 5). Calibration curves and DCA demonstrated good agreement and clinical net benefit in internal validation. However, external net benefit varied across thresholds, indicating limited generalizability (Supplementary Fig. 3). Time-dependent ROC analysis further confirmed stable prediction performance across 1, 2, and 3 year OS (Supplementary Fig. 4).

**Table 3:** The *C*-index values of each model in the training, internal validation, and external validation cohorts

Models	Training cohort	Internal validation cohort	External validation cohort
	*C*-index (95% CI)	*C*-index (95% CI)	*C*-index (95% CI)
Clinical model	0.707 (95% CI: 0.664–0.750)	0.687 (95% CI: 0.627–0.748)	0.645 (95% CI: 0.539–0.752)
DL model	0.781 (95% CI: 0.747–0.815)	0.743 (95% CI: 0.683–0.803)	0.692 (95% CI: 0.576–0.809)
Combined model	0.803 (95% CI: 0.770–0.836)	0.753 (95% CI: 0.697–0.808)	0.725 (95% CI: 0.613–0.838)

### DL model reveals metabolic pathway heterogeneity in ESCC

**Fig. 6:**
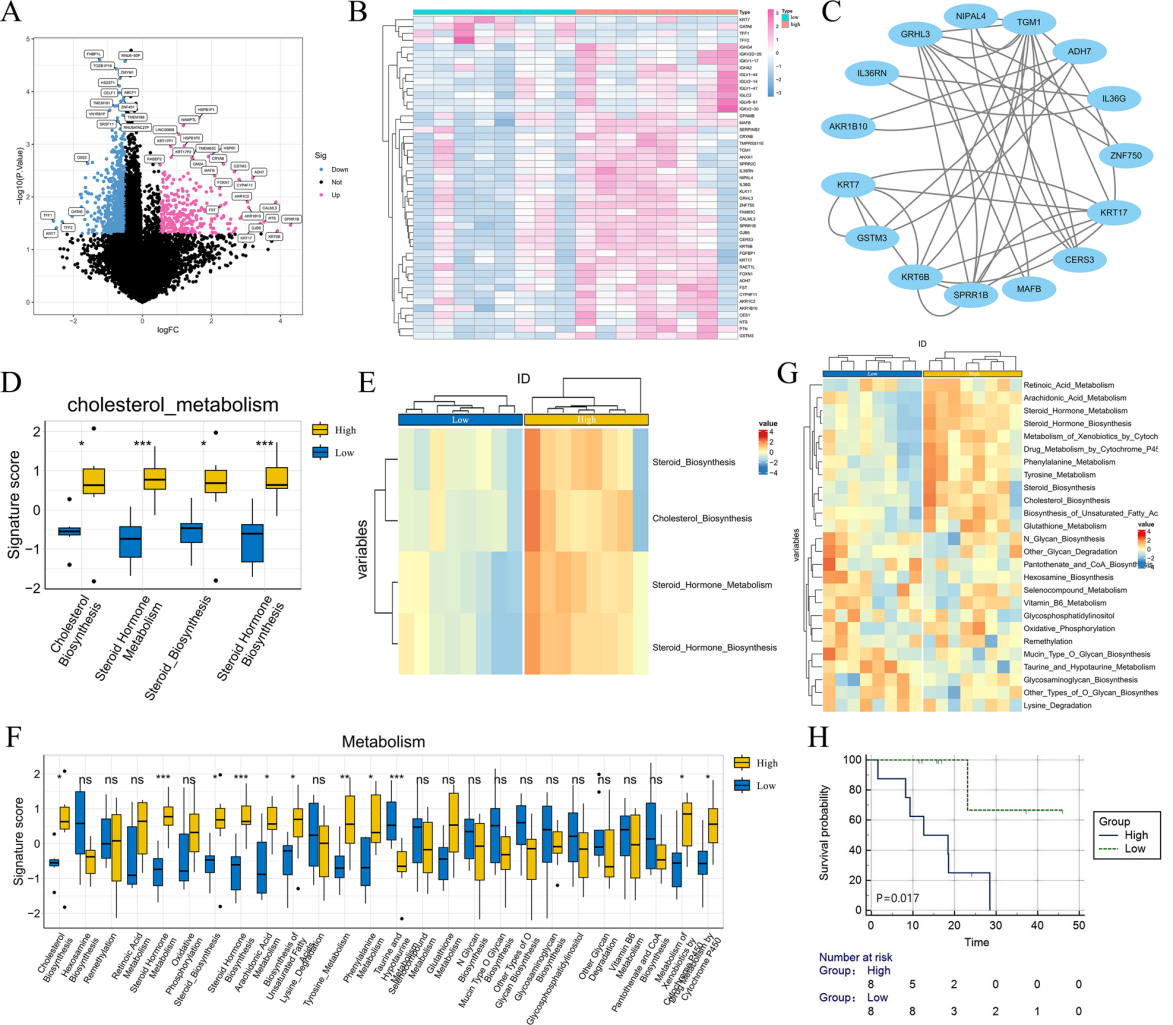
Transcriptomic analysis of metabolic heterogeneity between radiomics-defined risk groups in ESCC. **A** Volcano plot showing DEGs between high- and low-risk groups defined by DLS. **B** Heatmap of top DEGs showing distinct gene expression patterns. **C** PPI network of representative DEGs. **D**–**F** Metabolic pathway analysis showing higher activity of cholesterol and steroid hormone biosynthesis in the high-risk group. **G** Heatmap of ssGSEA scores across multiple metabolism-related pathways. **H** KM survival curve showing OS difference between transcriptomic subgroups (*p* = 0.017)

An exploratory transcriptomic analysis was conducted on 16 TCGA-ESCC patients, stratified into high- and low-risk groups based on the DL model. DEGs revealed distinct transcriptomic profiles. Upregulated genes in the high-risk group, including KRT17, AKR1B10, and GSTM3, were enriched in keratinization, redox metabolism, and inflammatory pathways, suggesting metabolic reprogramming and epithelial remodeling. A PPI network showed tightly connected functional modules centered around these genes. ssGSEA on curated metabolic gene sets indicated that the high-risk group exhibited significantly higher activity in cholesterol biosynthesis, steroid hormone metabolism, oxidative phosphorylation, and glutathione metabolism. These results were visualized via enrichment heatmaps, demonstrating distinct metabolic activity and clustering patterns in the high-risk subgroup (Fig. 6). Additional immune-related analyses (e.g., tumor immune estimation resource and immune exclusion) showed no significant differences between groups but are presented in Supplementary Figs. 5 and 6 for completeness. In summary, DLS-defined high-risk ESCC patients exhibited transcriptomic features indicative of enhanced lipid metabolism, oxidative stress regulation, and aggressive molecular phenotypes, providing mechanistic insights into imaging-based risk stratification.

## Discussion

In this study, we developed a multimodal predictive model integrating CT-based DL features and preoperative nutritional indicators to estimate 3-year OS in patients with ESCC, and systematically explored the transcriptomic basis underlying the DL model. The results demonstrated that the combined model outperformed both the clinical and DL models across multiple validation cohorts. Furthermore, patients in the high-risk subgroup, as defined by the DLS risk scores, exhibited metabolic reprogramming characterized by enhanced activity in several key metabolic pathways. These findings provide a biologically interpretable, noninvasive prognostic tool for ESCC patients, while also offering novel insights into the potential link between imaging phenotypes and tumor metabolic heterogeneity.

The DL model developed in this study exhibited favorable predictive performance in both internal and external validation cohorts, with a *C*-index of 0.743 (95% CI: 0.683–0.803) and 0.692 (95% CI: 0.576–0.809), respectively. To further explore the underlying decision-making process of the model, we visualized the attention maps generated by the Crossformer architecture (Supplementary Fig. 7). The attention maps demonstrate that the model consistently focuses on tumor subregions with moderate to high signal intensity on the grayscale images, rather than the darkest zones. These may correspond to enhancing components, viable tumor cores, or structural interfaces, suggesting the model prioritizes biologically active or morphologically distinct areas. This visualization provides an intuitive understanding of the model’s focus and contributes to improved interpretability of the deep learning framework.

Metabolic reprogramming is increasingly recognized as a hallmark of cancer progression. In ESCC, tumor cells leverage enhanced lipid synthesis, oxidative phosphorylation, and glutathione metabolism to sustain growth and resist apoptosis [20, 21]. In this study, TCGA-ESCC patients with higher DLS exhibited significantly elevated activity in cholesterol biosynthesis, steroid metabolism, and redox-related pathways (*p* < 0.05), reflecting aggressive tumor phenotypes and enhanced antioxidant capacity [22, 23]. DEGs analysis further identified enrichment of metabolism-related enzymes and keratin family genes (e.g., KRT17, AKR1B10, GSTM3) in the high-risk group, supporting active metabolic remodeling [24, 25]. PPI analysis revealed tightly connected modules associated with keratinization and redox regulation. Notably, prior studies have shown that radiomic features may capture metabolic or immune states. Lin [26] demonstrated that an MRI-based model in nasopharyngeal carcinoma reflected interferon activity and T-cell infiltration. Wang [27] linked a CT-based radiomics signature to transcriptomic TACE response in hepatocellular carcinoma. Grossmann [28] reported radiomics-immune associations in lung cancer. While further validation is needed, our findings suggest that DL-derived imaging features may serve as a “digital biopsy,” reflecting tumor metabolic adaptation to hypoxia and oxidative stress. Compared to traditional radiomic approaches that rely on handcrafted features, our deep learning-based model leverages automatically extracted high-dimensional representations from raw CT data. The ability of DLS to capture transcriptomic-level metabolic heterogeneity without explicit feature engineering suggests a broader applicability and stronger potential.

In addition to the DL model, a clinical model was constructed in this study based on independent prognostic factors. Variables included in this model—such as pathological T stage, pathological N stage, perineural invasion, and lymphovascular invasion—reflect the local aggressiveness of the tumor and its invasive potential into surrounding tissues, indicating a higher risk of poor prognosis [29–31]. Moreover, LSMM, a marker of nutritional deficiency and systemic inflammation, was an independent predictor of OS and significantly improved the combined model’s stratification performance. Prior studies have shown that LSMM is associated with impaired immunity, poor treatment tolerance, and worse outcomes in ESCC and other malignancies [32–34]. Its integration allowed the model to incorporate host-related vulnerability, extending risk assessment beyond tumor-centric factors. In the training and internal validation cohorts, KM survival analysis demonstrated significant discrimination between high- and low-risk groups (*P* < 0.0001 in both cohorts), indicating the robustness of the clinical model within the development dataset. However, in the external validation cohort, no statistically significant difference in OS was observed between the two groups (*P* = 0.22), potentially reflecting the limited generalizability of the model to independent patient populations. This discrepancy may be attributed to heterogeneity in patient characteristics, treatment protocols, or sample size differences in the external cohort.

Compared with previous prognostic models based solely on clinicopathological features or handcrafted radiomic features, our combined model demonstrated superior performance and broader clinical applicability. For example, Zhang [35] developed a clinical model using SEER data with a *C*-index of approximately 0.609, but it lacked molecular or imaging biomarkers and showed limited generalizability. Huang [36] proposed a deep learning model based on CT images to predict outcomes in ESCC patients undergoing immunochemotherapy. Although it achieved moderate predictive power (*C*-index ~ 0.74 in internal and ~0.61 in external cohorts), it did not incorporate key clinical or nutritional variables, which may have compromised robustness. In contrast, our model integrates DL-based imaging features, independent clinical prognostic factors, and nutritional status, yielding higher *C*-index values of 0.753 and 0.725 in internal and external validations, respectively. This integrative approach enhances both predictive accuracy and biological interpretability, supporting its potential for clinical translation.

This study has limitations. First, as a retrospective multicenter analysis, it may be affected by selection bias and data heterogeneity. Second, the external validation sample was relatively small, warranting prospective validation in larger, more diverse cohorts. Third, follow-up duration was limited, and treatment data were recorded only as binary variables, referring specifically to postoperative adjuvant therapy (e.g., chemotherapy, radiotherapy, or chemoradiotherapy), thus lacking treatment-specific granularity. Fourth, due to the retrospective nature of the study and the absence of repeated scans or segmentation perturbations, we could not formally assess the reproducibility of DL features, which should be addressed in future prospective studies. Finally, although transcriptomic analysis revealed metabolic differences between risk groups, the small sample size and lack of experimental validation limit the mechanistic interpretation of these findings.

## Conclusion

We developed and validated a multimodal model to predict 3-year OS in ESCC patients. Risk stratification based on the DLS revealed metabolic pathway differences between subgroups, offering biological insight into imaging phenotypes. Future work should adopt multi-omics approaches to better understand tumor biology and improve the clinical interpretability and utility of predictive models.

## Supplementary information

Supplementary Material

## Data Availability

The datasets generated and/or analyzed during the current study are not publicly available because the subjects did not provide written consent for their data to be publicly shared.

## Abbreviations

*C*-index: Concordance index
DCA: Decision curve analysis
DEGs: Differentially expressed genes
ESCC: Esophageal squamous cell carcinoma
KM: Kaplan–Meier
LASSO: Least absolute shrinkage and selection operator
OS: Overall survival
PPI: Protein–protein interaction
SMI: Skeletal muscle index
ssGSEA: Single-sample gene set enrichment analysis
TCGA: The cancer genome atlas

## References

[1] Waters JK, Reznik SI (2022) Update on management of squamous cell esophageal cancer. Curr Oncol Rep 24:375–385. 10.1007/s11912-021-01153-4 35142974 10.1007/s11912-021-01153-4

[2] Bray F, Ferlay J, Soerjomataram I, Siegel RL, Torre LA, Jemal A (2018) Global cancer statistics 2018: GLOBOCAN estimates of incidence and mortality worldwide for 36 cancers in 185 countries. CA Cancer J Clin. 10.3322/caac.21492 10.3322/caac.21492 30207593

[3] Greene FL, Sobin LH (2008) The staging of cancer: a retrospective and prospective appraisal. CA Cancer J Clin. 10.3322/CA.2008.0001 10.3322/CA.2008.0001 18460593

[4] Mony JT, Schuchert MJ (2018) Prognostic implications of heterogeneity in intra-tumoral immune composition for recurrence in early stage lung cancer. Front Immunol. 10.3389/fimmu.2018.02298 10.3389/fimmu.2018.02298 PMC6196259 30374348

[5] González Santos S, Martí Gelonch L, González Jorrín N, González Osinalde M, Rosell Romero N (2024) Preoperative risk assessment and prehabilitation strategies in patients undergoing an esophagectomy for cancer resections: a single center retrospective analysis and a review of the literature. Front Anesthesiol 3:1358847

[6] Power S, Maarof A, Power A, Feehan S, Whelan M (2024) Nutritional risk predicts postoperative complications and length of stay, whereas sarcopenia risk predicts need for step-down care in a mixed elective surgery population. J Hum Nutr Diet 37:308–315 37908178 10.1111/jhn.13256

[7] Ida S, Kumagai K, Nunobe S (2022) Current status of perioperative nutritional intervention and exercise in gastric cancer surgery: a review. Ann Gastroenterol Surg 6:197–203 35261945 10.1002/ags3.12520 PMC8889851

[8] Jogiat UM, Baracos V, Turner SR et al (2023) Changes in sarcopenia status predict survival among patients with resectable esophageal cancer. Ann Surg Oncol 30:7412–7421 37466867 10.1245/s10434-023-13840-6

[9] Yan T, Yan Z, Chen G et al (2025) Survival outcome prediction of esophageal squamous cell carcinoma patients based on radiomics and mutation signature. Cancer Imaging 25:1–13 39891186 10.1186/s40644-024-00821-5 PMC11783911

[10] Luan J, Zhang D, Liu B et al (2024) Immune-related lncRNAs signature and radiomics signature predict the prognosis and immune microenvironment of glioblastoma multiforme. J Transl Med 22:107 38279111 10.1186/s12967-023-04823-y PMC10821572

[11] Fiz F, Rossi N, Langella S et al (2024) Radiomics of intrahepatic cholangiocarcinoma and peritumoral tissue predicts postoperative survival: development of a CT-based clinical-radiomic model. Ann Surg Oncol 31:5604–5614 38797789 10.1245/s10434-024-15457-9

[12] Peng H, Xue T, Chen Q, Li M, Ge Y, Feng F (2022) Computed tomography-based radiomics nomogram for predicting the postoperative prognosis of esophageal squamous cell carcinoma: a multicenter study. Acad Radiol 29:1631–1640 35300908 10.1016/j.acra.2022.01.020

[13] Kawahara D, Nishioka R, Murakami Y, Emoto Y, Iwashita K, Sasaki R (2024) A nomogram based on pretreatment radiomics and dosiomics features for predicting overall survival associated with esophageal squamous cell cancer. Eur J Surg Oncol 50:108450 38843660 10.1016/j.ejso.2024.108450

[14] Guo Z, Ma J, Zhang J et al (2025) Metabolic reprogramming and immunological changes in the microenvironment of esophageal cancer: future directions and prospects. Front Immunol 16:1524801 10.3389/fimmu.2025.1524801 PMC11802498 39925801

[15] Huang Q, Chen H, Yin D et al (2024) Multi-omics analysis reveals NNMT as a master metabolic regulator of metastasis in esophageal squamous cell carcinoma. npj Precis Oncol 8: 24 38291241 10.1038/s41698-024-00509-w PMC10828394

[16] Wang Y, Shi Y, Hu X, Wang C (2025) Targeting glycolysis in esophageal squamous cell carcinoma: single-cell and multi-omics insights for risk stratification and personalized therapy. Front Pharmacol 16:1559546 10.3389/fphar.2025.1559546 PMC11922847 40115255

[17] Wang Z, Zhang Y, Yang X et al (2024) Genetic and molecular characterization of metabolic pathway-based clusters in esophageal squamous cell carcinoma. Sci Rep 14:6200 38486026 10.1038/s41598-024-56391-w PMC10940668

[18] Mourtzakis M, Prado CMM, Lieffers JR, Reiman T, McCargar LJ, Baracos VE (2008) A practical and precise approach to quantification of body composition in cancer patients using computed tomography images acquired during routine care. Appl Physiol Nutr Metab 33:997–1006 18923576 10.1139/H08-075

[19] Cho WK, Yu JI, Park HC, Lim DH, Kim TH, Chie EK (2021) Impact of sarcopenia on survival of pancreatic cancer patients treated with concurrent chemoradiotherapy. Tumori 107:247–253 32646298 10.1177/0300891620937795

[20] Wang Z, Sun X, Li Z, Yu H, Li W, Xu Y (2024) Metabolic reprogramming in esophageal squamous cell carcinoma. Front Pharmacol 15:1423629 10.3389/fphar.2024.1423629 PMC11233760 38989149

[21] Xue X, Wang M, Cui J et al (2025) Glutathione metabolism in ferroptosis and cancer therapy. Cancer Lett 621:217697 10.1016/j.canlet.2025.217697 40189013

[22] Chen Y, Yang H, Huang X, Wang R, Mao W, Chen Z (2023) Metabolomics and transcriptomics joint analysis reveals altered amino acid metabolism in esophageal squamous cell carcinoma. Research Square rs-3117927/v1. 10.21203/rs.3.rs-3117927/v1

[23] Jin X, Liu L, Wu J et al (2021) A multi-omics study delineates new molecular features and therapeutic targets for esophageal squamous cell carcinoma. Clin Transl Med 11:e538 34586744 10.1002/ctm2.538 PMC8473482

[24] Jin X, Liu L, Liu D et al (2024) Unveiling the methionine cycle: a key metabolic signature and NR4A2 as a methionine-responsive oncogene in esophageal squamous cell carcinoma. Cell Death Differ 31:558–573 38570607 10.1038/s41418-024-01285-7 PMC11094133

[25] Li S, Huang W, Chen Y et al (2024) Aldo-keto reductase family 1 member B10 prevents esophageal squamous cell carcinoma from reactive carbonyl species-induced cell death and promotes its progression. Cancer Cell Int 24:425 39710692 10.1186/s12935-024-03623-8 PMC11663324

[26] Lin D, Li H, Liu T et al (2024) Radiomic signatures associated with tumor immune heterogeneity predict survival in locally recurrent nasopharyngeal carcinoma. J Natl Cancer Inst 116:1294–1302 38637942 10.1093/jnci/djae081

[27] Wang C, Leng B, You R et al (2024) A transcriptomic biomarker for predicting the response to TACE correlates with the tumor microenvironment and radiomics features in hepatocellular carcinoma. J Hepatocell Carcinoma 11:2321–2337 10.2147/JHC.S480540 PMC11606151 39619603

[28] Grossmann P, Stringfield O, El-Hachem N et al (2017) Defining the biological basis of radiomic phenotypes in lung cancer. eLife 16:6e23421 10.7554/eLife.23421 PMC5590809 28731408

[29] Huang Q, Luo K, Chen C et al (2016) Identification and validation of lymphovascular invasion as a prognostic and staging factor in node-negative esophageal squamous cell carcinoma. J Thorac Oncol 11:583–592 26792626 10.1016/j.jtho.2015.12.109

[30] Chen J, Xie J, Ling Y et al (2014) The prognostic effect of perineural invasion in esophageal squamous cell carcinoma. BMC Cancer 14:313 10.1186/1471-2407-14-313 PMC4016635 24886020

[31] Zhou J, Yang Y, Zhang H et al (2023) Lymphovascular and perineural invasion after neoadjuvant therapy in esophageal squamous carcinoma. Ann Thorac Surg115:1386–1394 36027933 10.1016/j.athoracsur.2022.07.052

[32] Wakefield CJ (2023) Editorial comment on “Changes in Sarcopenia Status Predict Survival Among Patients with Resectable Esophageal Cancer”. Ann Surg Oncol. 10.1245/s10434-023-14047-5 10.1245/s10434-023-14047-5 37610491

[33] Mallet R, Modzelewski R, Lequesne J, Mihailescu SD, Thureau S (2020) Prognostic value of sarcopenia in patients treated by radiochemotherapy for locally advanced oesophageal cancer. Radiat Oncol. 10.21203/rs.2.23302/v3 10.1186/s13014-020-01545-z PMC7245030 32443967

[34] Cho WK, Yu JI, Park HC, Lim DH, Chie EK (2020) Impact of sarcopenia on survival of pancreatic cancer patients treated with concurrent chemoradiotherapy. Tumori. 10.1177/0300891620937795 10.1177/0300891620937795 32646298

[35] Zhang M, Cui M, Zuo Q et al (2021) Construction and evaluation of prognostic models for esophageal cancer patients with distant and non-distant metastases: providing a reference process for clinical diagnosis and treatment. J Gastrointest Oncol 12:1241 34532084 10.21037/jgo-21-429 PMC8421894

[36] Huang X, Huang Y, Li P, Xu K (2025) CT-based deep learning predicts prognosis in esophageal squamous cell cancer patients receiving immunotherapy combined with chemotherapy. Acad Radiol 32:3397–3409 10.1016/j.acra.2025.01.046 39956748

