# Supplementary material for: CT-based deep learning signatures associated with transcriptomic heterogeneity and combined with nutritional biomarkers improve prediction of 3-year overall survival in esophageal squamous cell carcinoma
Source: Insights Imaging. 2026 Jan 26;17:22. doi: 10.1186/s13244-025-02189-x (PMC12835474; doi:10.1186/s13244-025-02189-x)
Supplement: Supplementary file 1 — Supplementary Material [file 13244_2025_2189_MOESM1_ESM.pdf]

CT-Based Deep Learning Signatures Associated with Transcriptomic Heterogeneity and Combined with Nutritional Biomarkers Improve Prediction of 3-Year Overall Survival in Esophageal Squamous Cell Carcinoma

ELECTRONIC SUPPLEMENTARY MATERIAL

Supplementary Material 1: Detailed definitions of low skeletal muscle mass (LSMM)

The skeletal muscle index (SMI) was calculated as skeletal muscle area divided by height squared (SMI = skeletal muscle area / height^2, cm^2/m^2). LSMM was defined as an SMI < 36.2 cm^2/m^2 for males or < 29.6 cm^2/m^2 for females, and was used to evaluate patients' nutritional status and its association with prognosis. The cutoff values for skeletal muscle index (SMI) in this study were adopted from previously published LSMM literature. However, it should be noted that, according to international consensus, the diagnosis of LSMM additionally requires the assessment of muscle strength and/or physical performance. As our analysis was based solely on CT-derived SMI, the results in this study strictly refer to "low skeletal muscle mass (LSMM)" as defined by these thresholds, rather than a definitive diagnosis of sarcopenia.

Supplementary Material 2: Contrast-enhanced CT Acquisition Parameters

Scans were performed on Siemens SOMATOM Force and SOMATOM Definition scanners during a single breath-hold, from the thoracic inlet to the lung bases. Standard parameters were: tube voltage 100–120 kV and tube current ~300 mA; helical acquisition with a pitch of approximately 1.0 (range 0.8–1.2, adjusted by patient habitus and automatic exposure control); reconstruction matrix 512 × 512; and a medium soft-tissue reconstruction kernel (Siemens B30f/B31f or equivalent). Slice thickness was 5 mm for clinical viewing. A nonionic contrast agent (iopamidol) was injected via the antecubital vein at 1.5 mL/kg with a flow rate of 3 mL/s, and venous-phase imaging was acquired with a 50-s delay.

Supplementary Figure S1: Results of Univariate Cox Regression Analysis

Variable	Estimate	S.E	HR (95%CI)	Z	P
Gender					
Female	Ref				
Male	0.309	0.186	1.363 (0.946, 1.962)	1.662	0.096
Age					
> 60	Ref				
≤60	−0.096	0.168	0.908 (0.654, 1.261)	−0.575	0.566
Location					
Middle	Ref				
Lower	0.186	0.184	1.204 (0.840, 1.727)	1.012	0.312
Upper	−0.258	0.267	0.773 (0.458, 1.304)	−0.965	0.334
Differentiation					
Poor	Ref				
Moderate	−0.284	0.221	0.753 (0.488, 1.161)	−1.283	0.200
Well	−0.452	0.246	0.636 (0.393, 1.032)	−1.833	0.067
Type					
Ulcerative	Ref				
Medullary	0.224	0.183	1.251 (0.874, 1.790)	1.222	0.222
Fungating	−0.433	0.421	0.649 (0.284, 1.481)	−1.027	0.304
Stenotic	−0.304	1.005	0.738 (0.103, 5.294)	−0.302	0.762
Neuroaggression					
No	Ref				
Yes	0.955	0.242	2.600 (1.619, 4.174)	3.954	<0.001*
Vascular_invasion					
No	Ref				
Yes	0.712	0.181	2.037 (1.429, 2.906)	3.929	<0.001*
pT					
2	Ref				
3	1.025	0.202	2.787 (1.875, 4.144)	5.068	<0.001*
4	0.801	0.256	2.228 (1.348, 3.680)	3.126	0.002*
pN					
0	Ref				
1	0.640	0.197	1.896 (1.288, 2.791)	3.245	0.001*
2	1.419	0.225	4.135 (2.663, 6.421)	6.322	<0.001*
3	1.875	0.383	6.521 (3.080, 13.806)	4.900	<0.001*
Therapy					
Yes	Ref				
No	−0.819	0.170	0.441 (0.316, 0.615)	−4.822	<0.001*
Sarcopenia					
No	Ref				
Yes	0.388	0.177	1.474 (1.042, 2.085)	2.195	0.028*
Muscular_steatosis					
Yes	Ref				
No	0.058	0.170	1.059 (0.758, 1.479)	0.338	0.735
IMAT	−0.023	0.016	0.977 (0.948, 1.008)	−1.475	0.140
VAT	0.000	0.002	1.000 (0.997, 1.003)	0.074	0.941
SAT	−0.003	0.002	0.997 (0.994, 1.000)	−1.863	0.062
Height	0.011	0.011	1.012 (0.989, 1.034)	1.009	0.313
Weight	−0.003	0.009	0.997 (0.980, 1.014)	−0.317	0.751

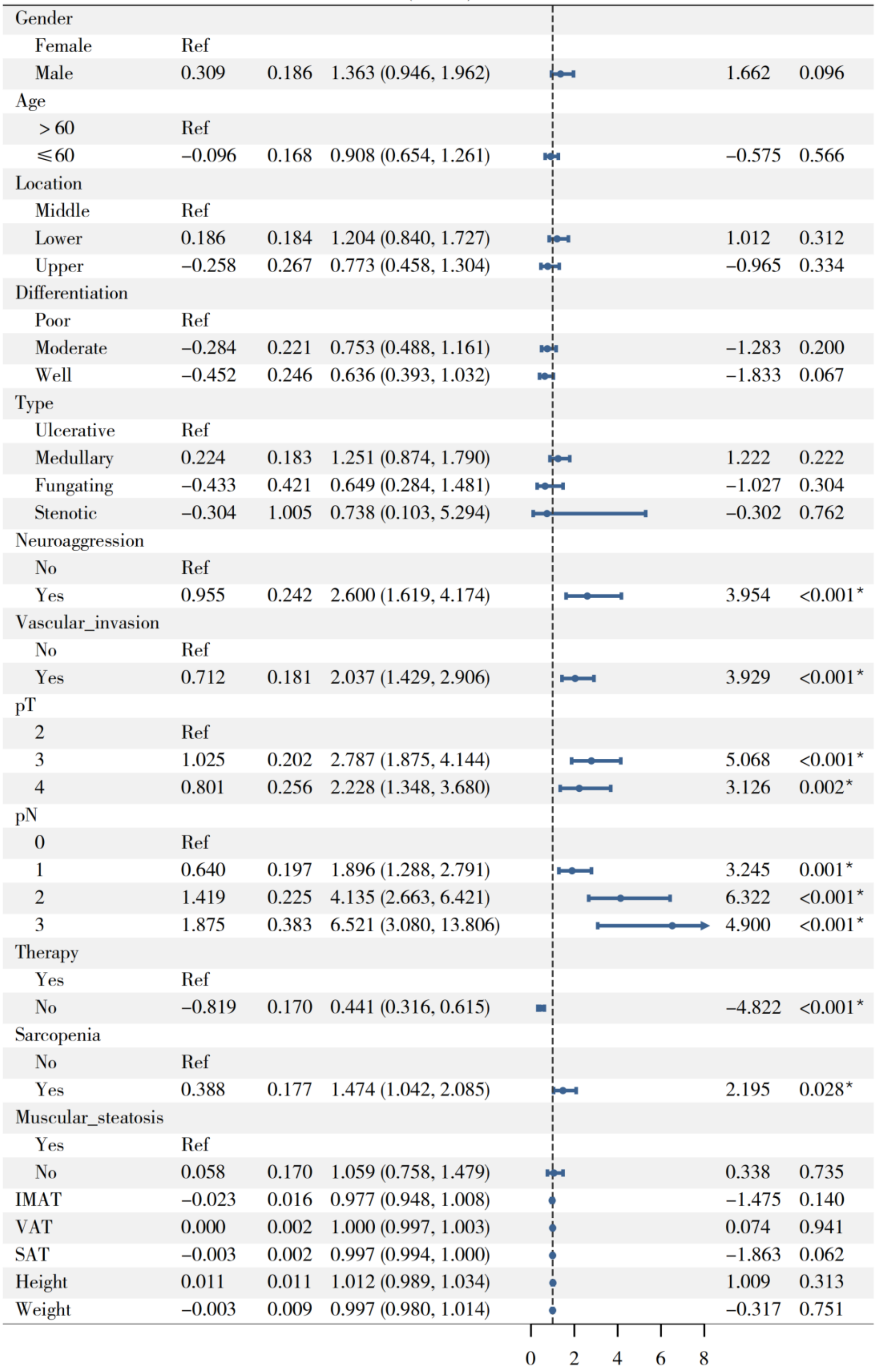

Supplementary Figure S2: DL Features Selected for Model Building

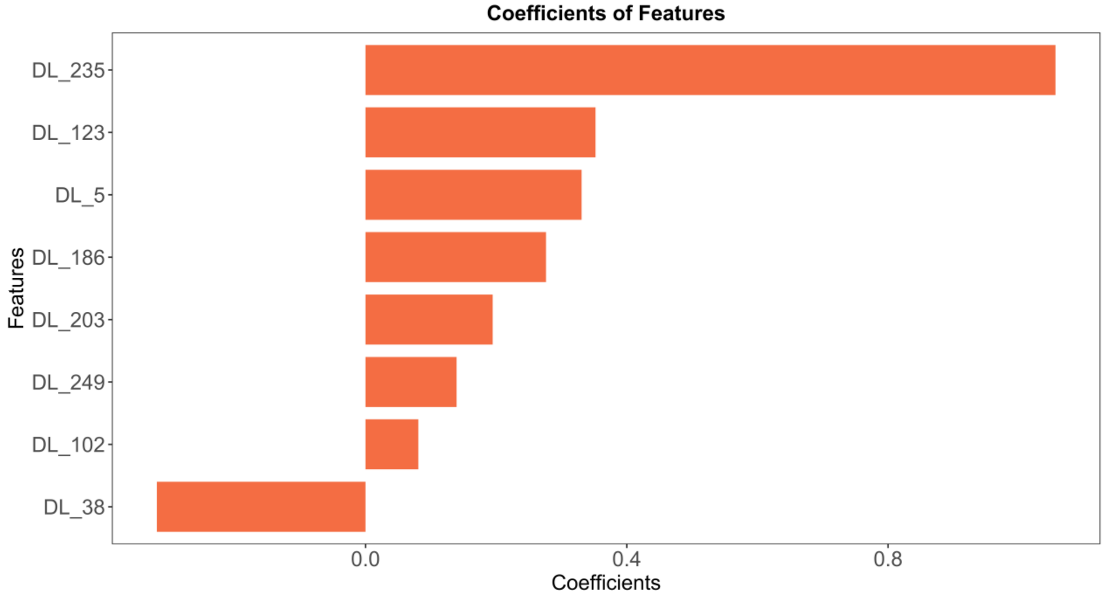

Supplementary Figure S3: Calibration curves and DCA curves

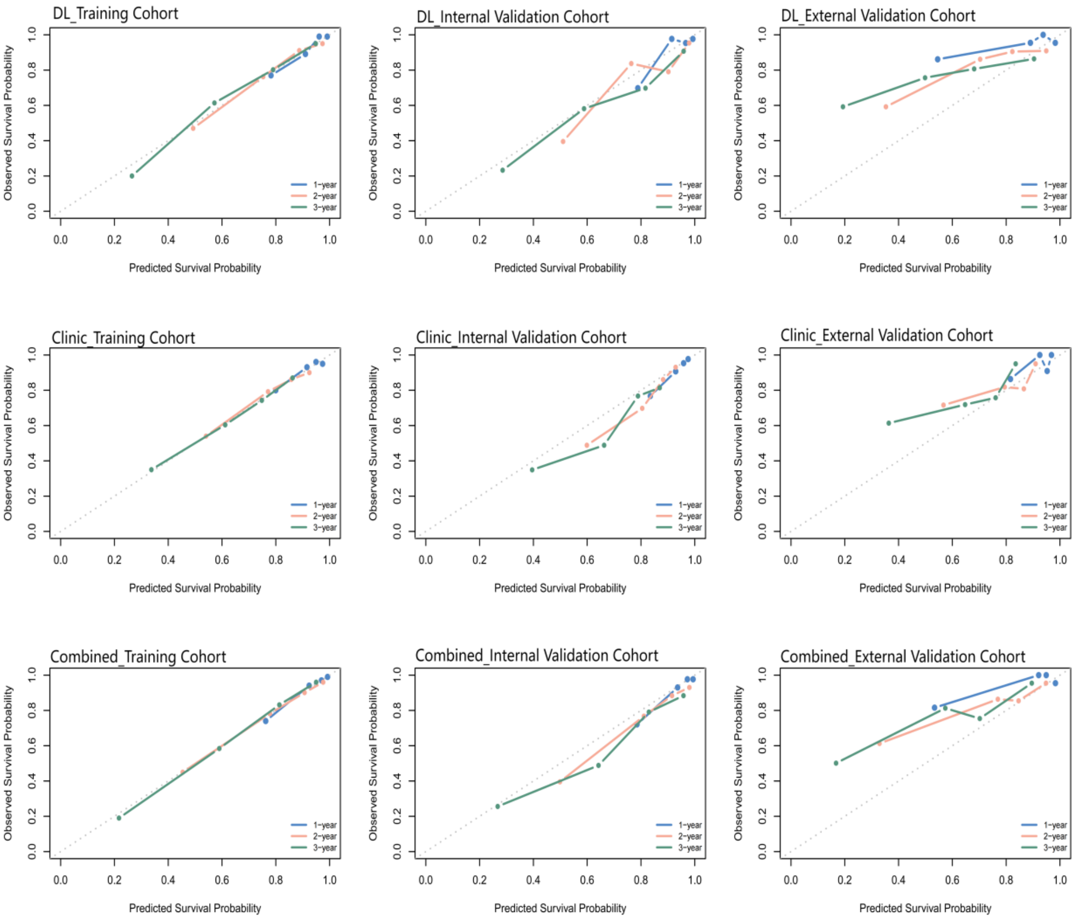

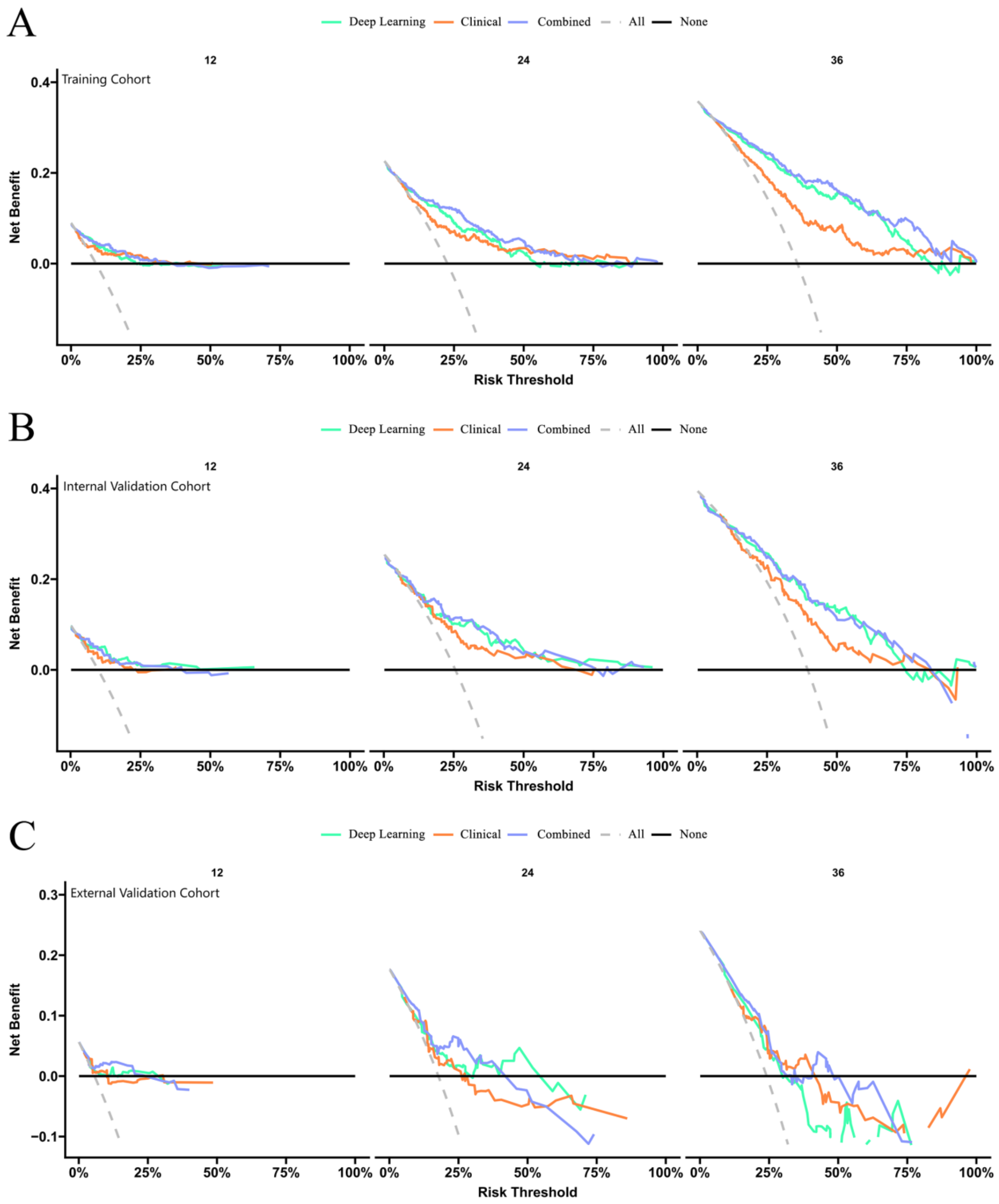
A
Deep Learning
Clinical
Combined
All
None
12
24
36
Training Cohort
Net Benefit
0.4
0.2
0.0
0%
25%
50%
75%
100%
Risk Threshold
B
Internal Validation Cohort
C
External Validation Cohort
0.3
0.1
−0.1

.

Supplementary Figure S4: Time-Dependent ROC Curves

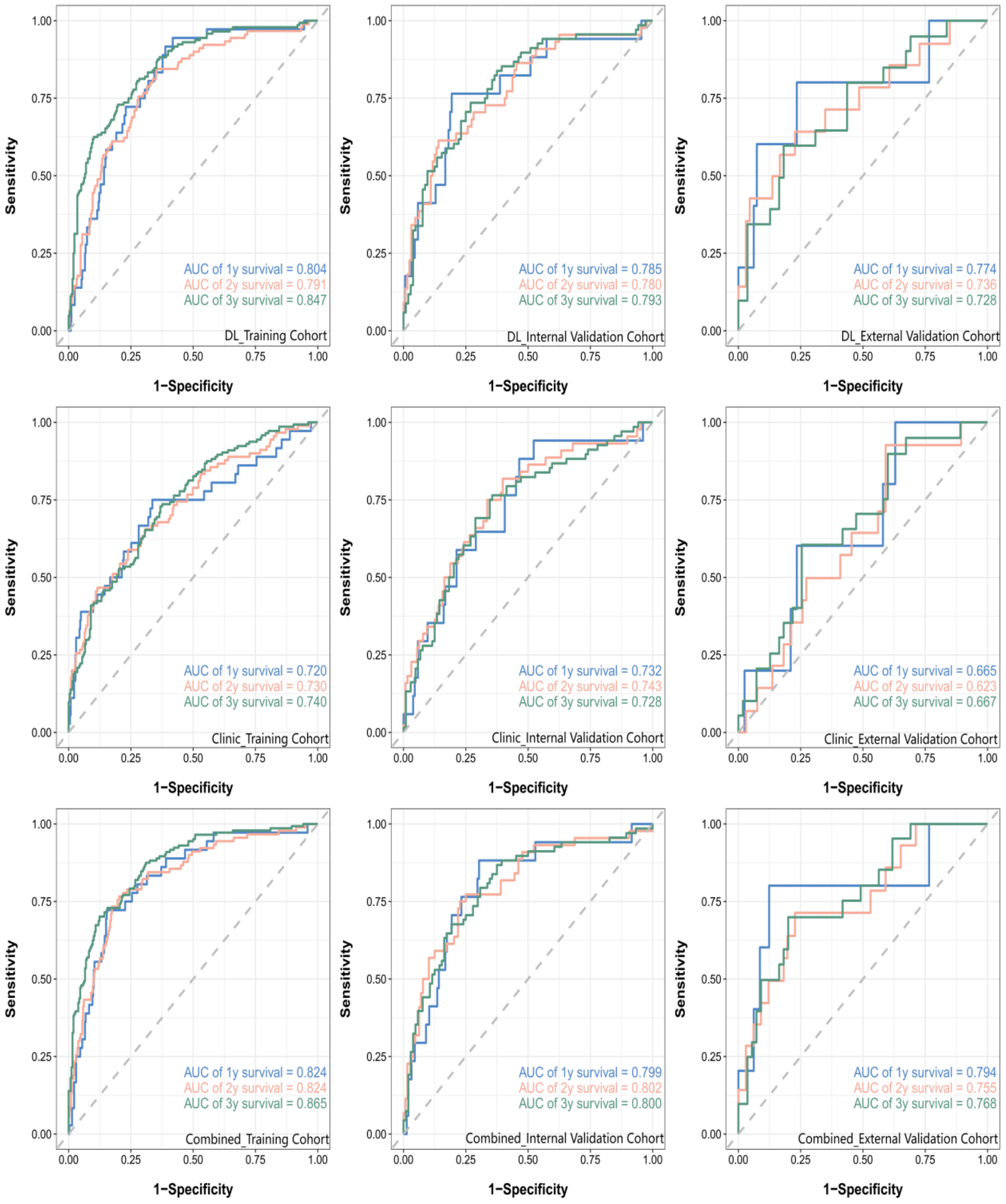

Supplementary Figure S5: Comparison of immune-related signatures between high and low groups. (A) Differences in the immune microenvironment signatures between high and low groups, including GPAGs, PPAGs, Pan_F_TBRs, and multiple published immune-related scores. (B) Immune exclusion signatures analyzed across groups, such as CAF, fibroblast, TGFβ-related, and macrophage exclusion scores. (C–D) Two panels of tumor microenvironment-related gene signatures (tme_signatures1 and tme_signatures2) comparing gene expression patterns in high vs. low groups, highlighting differences in T cell exhaustion, regulatory T cell, and macrophage-related pathways. (E) Immune cell infiltration levels derived from TIMER analysis, including B cells, CD4+ and CD8+ T cells, neutrophils, macrophages, and dendritic cells.

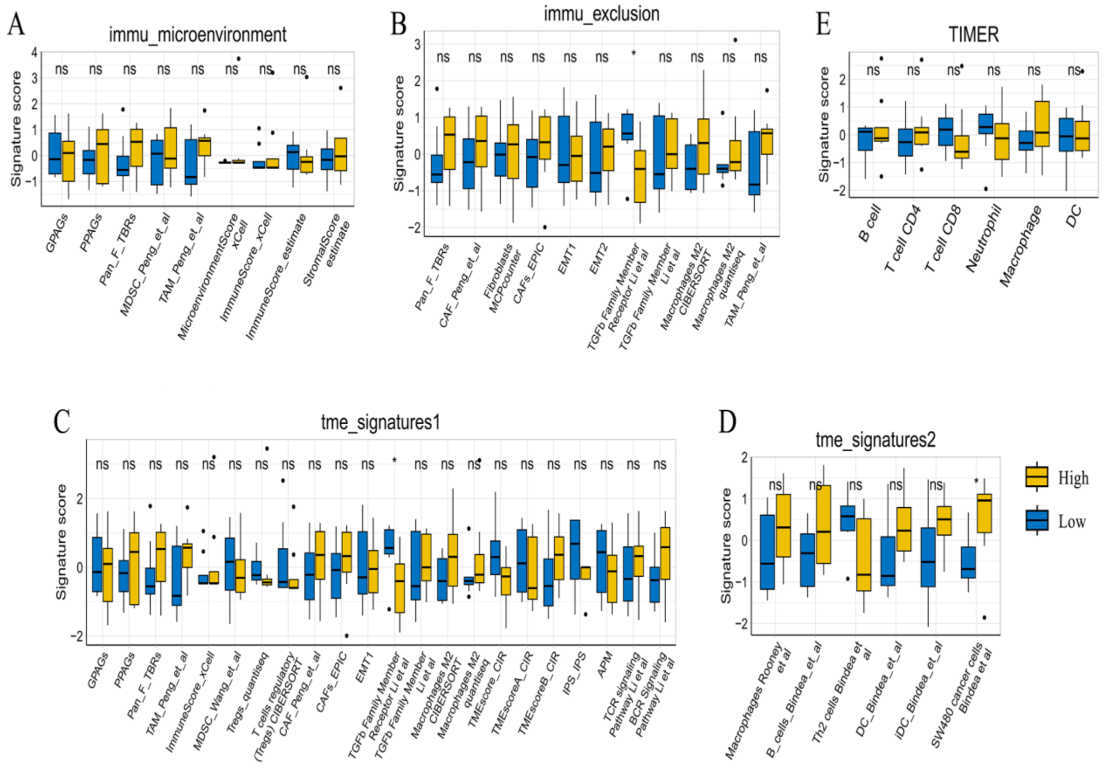

Supplementary Figure S6: Heatmap analysis of immune and stromal-related signatures corresponding to Supplementary 5. (A–E) Heatmaps displaying the expression patterns of the same sets of signatures shown in Supplementary Figure SS4 panels A–E, respectively. Each heatmap illustrates hierarchical clustering of samples (columns) and signature scores (rows), with the top annotation indicating group classification (Low: blue; High: yellow). (A) Immune microenvironment signatures. (B) Immune exclusion-related signatures. (C–D) Two panels of tumor microenvironment-related gene signatures (tme_signatures1 and tme_signatures2). (E) Immune cell infiltration profiles from TIMER.

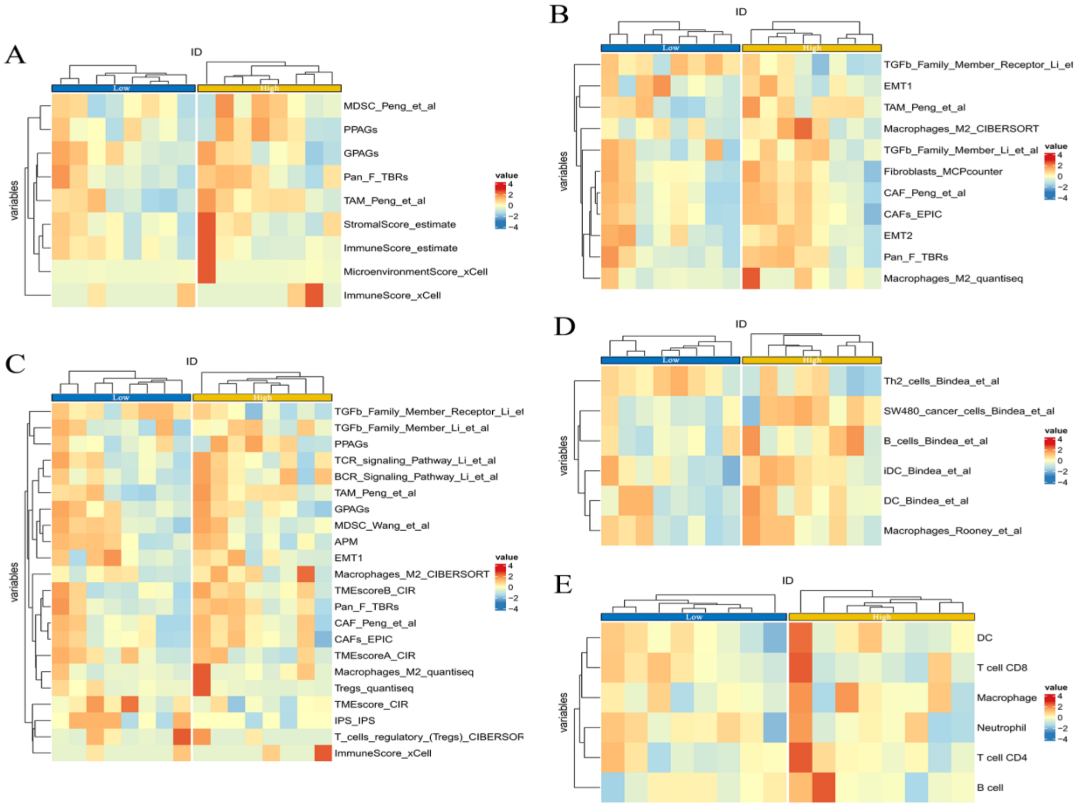

Supplementary Figure S7: Attention maps (left) and corresponding grayscale tumor slices (right) from the largest tumor axial slice and its adjacent upper and lower layers in a representative case. The attention maps were generated by the Crossformer model and visualize spatial regions within the tumor that receive higher attention during survival prediction. High-attention areas (red to yellow) are observed to cluster around distinct subregions and demonstrate consistency across adjacent slices. This visualization improves interpretability by highlighting how the model attends to tumor heterogeneity in three contiguous layers centered around the largest lesion cross-section.

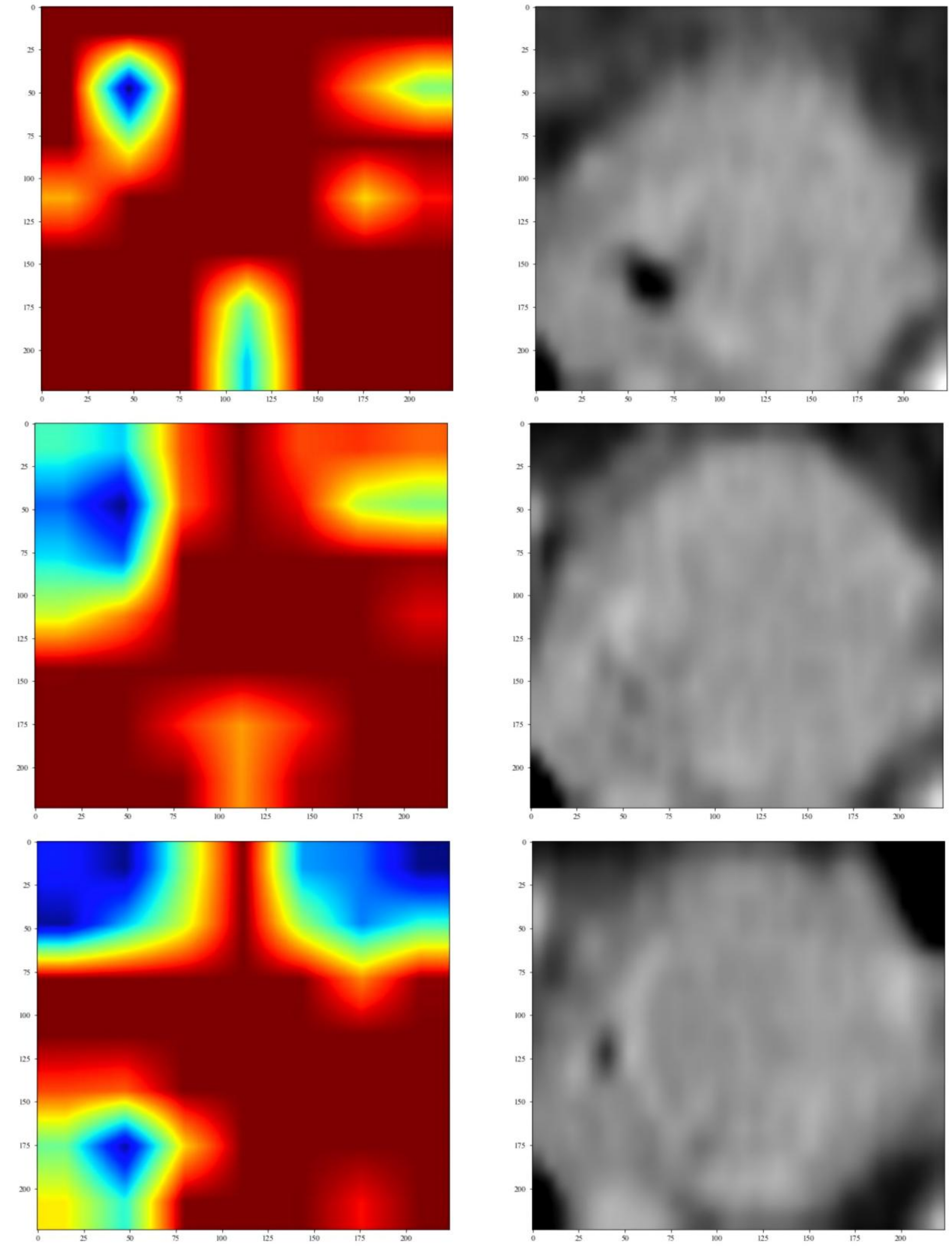

Supplementary Table S1: Key Hyper-Parameters, Preprocessing Settings, and Reproducibility Information for the Crossformer-based Deep Learning Model

Category	Item	Value/Setting
Backbone	Model	Crossformer
	Depth / Heads / Embedding dim / MLP dim / Patch size	6 / 16 / 1024 / 768 / 64
	Activation / Dropout	GELU / 0.1
Pretraining	Source	ImageNet classification weights
Optimization	Optimizer	Stochastic Gradient Descent (SGD)
	Initial learning rate	0.01
	Momentum / Weight decay	0.9 / 1e−4
	Batch size / Epochs	32 / 300
	Learning rate scheduler	None
Preprocessing	Resampling voxel size	1.0 × 1.0 × 1.0 mm
	Intensity window	−100 to 300 HU
	Normalization	ImageNet mean = [0.485, 0.456, 0.406], std = [0.229, 0.224, 0.225] (after replicating single channel to 3 channels)
Feature extraction	Hook layer	avgpool (global pooled embedding, 1024-d)
Dimensionality reduction	Method / Target dim	PCA / 256 (95.2% variance retained)
Feature selection	Method	mRMR filtering → LASSO-Cox regression
	Number of features	8

	selected	

Supplementary Table S2: The Dice coefficient was computed for 10 representative cases to illustrate inter-reader agreement. The calculation code is also provided.

Case_ID	Dice_Score
001.nii.gz	0.836
002.nii.gz	0.877
003.nii.gz	0.861
004.nii.gz	0.852
005.nii.gz	0.821
006.nii.gz	0.821
007.nii.gz	0.814
008.nii.gz	0.871
009.nii.gz	0.852
010.nii.gz	0.860
Mean±SD	0.846±0.022

```
import nibabel as nib
import numpy as np

def dice_coefficient(mask1_path, mask2_path):
    mask1_img = nib.load(mask1_path).get_fdata()
    mask2_img = nib.load(mask2_path).get_fdata()

    mask1_bin = mask1_img > 0
    mask2_bin = mask2_img > 0

    intersection = np.logical_and(mask1_bin, mask2_bin).sum()
    size1 = mask1_bin.sum()
    size2 = mask2_bin.sum()

    if size1 + size2 == 0:
        return 1.0

    dice = 2.0 * intersection / (size1 + size2)
    return dice
```

Supplementary Table S3: Prognostic impact of individual deep learning (DL) features assessed by Cox regression

Features	P	HR (95%CI)
DL_102	<0.0001	1.2456 (1.1307-1.3722)
DL_123	<0.0001	1.4856 (1.2550-1.7585)
DL_186	0.0097	1.2429 (1.0541-1.4656)
DL_203	<0.0001	1.3918 (1.2423-1.5594)
DL_235	<0.0001	3.8154 (2.2044-6.6040)
DL_249	<0.0001	1.1904 (1.1050-1.2824)
DL_38	<0.0001	0.6999 (0.6016-0.8143)
DL_5	<0.0001	1.5297 (1.3317-1.7572)
